# A new anatomic locking plate for the treatment of posterolateral tibial plateau fractures

**DOI:** 10.1186/s12891-018-2216-2

**Published:** 2018-09-05

**Authors:** Zhen Jian, Rongguang Ao, Jianhua Zhou, Xinhua Jiang, Dianying Zhang, Baoqing Yu

**Affiliations:** 1 Department of Orthopaedics, Shanghai Pudong Hospital, Shanghai Fudan Univercity Pudong Medical Center, Shanghai, 201399 China; 2 Department of Orthopedics, Peking University People’s Hospital, Beijing, 100044 China

**Keywords:** Posterolateral tibial plateau fractures, Anatomic locking plate, Posterolateral approach

## Abstract

**Background:**

Posterolateral tibial plateau fractures have become more common, and their treatment is of great importance to knee function. Additionally, there is no available literature detailing specialized anatomic locking plate for tibial plateau fractures. Therefore, the aim of the study was to evaluate the safety and clinical efficacy of an innovative anatomic locking plate for treatment of posterolateral tibial plateau fractures.

**Methods:**

Between March 2014 and January 2016, 12 patients with posterolateral tibial plateau fracture underwent surgery with the anatomic locking plate for the posterolateral tibial plateau via the posterolateral approach. Relevant operational data for clinical evaluation were collected.

**Results:**

The mean follow-up time was 26 months, and the mean age was 35 years for 12 patients. The mean interval between the time of injury and the surgery was 6.1 days. Radiological fracture union was evident in all patients at 12 weeks. During surgery, the blood loss ranged from 100 to 300 mL, and the duration ranged from 55 to 90 min. The Tegner–Lysholm functional score ranged from 85 to 97 at the final follow-up. Moreover, the final Rasmussen functional score ranged from 25 to 29, and Rasmussen anatomical score ranged from 13 to 18.

**Conclusions:**

The newly designed anatomic locking plate for the posterolateral tibial plateau provided adequate fixation along the posterolateral tibial plateau. It proved to be safe and effective in a small-sample-size population (12 patients) during a 12- to 34-month follow-up.

## Background

Tibial plateau fractures are commonly encountered in clinical practice, whereas posterolateral tibial plateau fractures have rarely been reported in the past. However, posterolateral tibial plateau fractures have been reported in up to 7% of all tibial plateau fractures with increased high-energy trauma and use of computed tomography (CT) [1–3].

Good reduction and firm fixation are vital for treatment of posterolateral tibial plateau fractures. Many authors recommend surgical treatment to restore joint congruity and mechanical stability and further guarantee the best prognosis [4, 5]. However, the operative treatment of posterolateral tibial plateau fractures remains a challenge due to the fibular head and ligamentous structures that impede adequate exposure of the posterolateral joint surface. Although many approaches have been reported [4, 6–9] to achieve appropriate exposure, there is no general consensus.

**Fig. 1:**
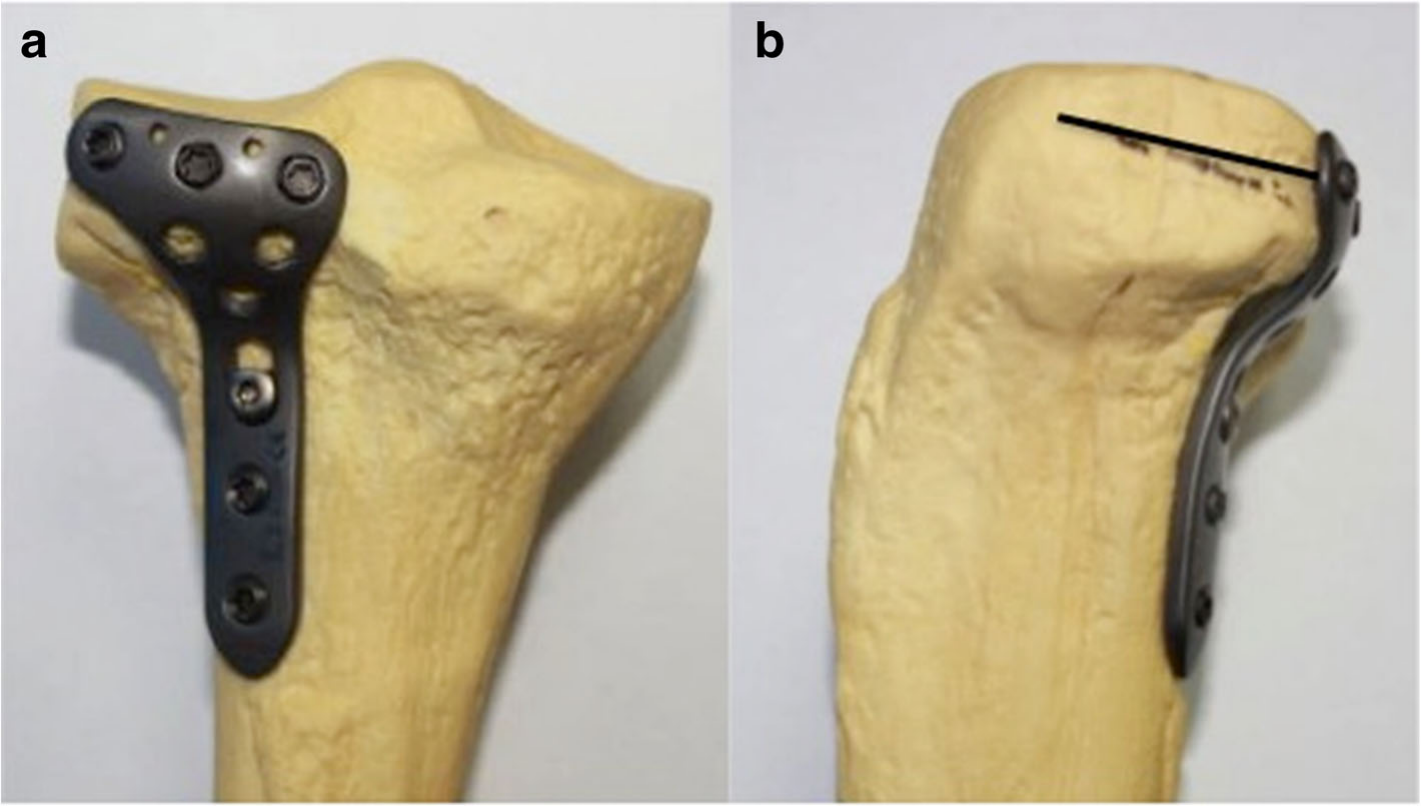
The newly designed posterolateral anatomic locking plate which is specially used in posterolateral tibial plateau fracture. **a**, posterior view of the tibial plateau. **b**, lateral view of tibial plateau

Recent studies [7–9] on the treatment of posterolateral tibial plateau fractures have focused on surgical approaches. There is no literature to our knowledge about a specialized anatomic locking plate for posterolateral tibial plateau fractures. Surgeons usually have to choose buttress plate systems such as L-shaped plates and T-plates used in the fixation of distal radius. However, due to the irregular bony structure of the region, the plates often require contouring during surgery to fit the posterolateral tibial plateau, which may potentially lead to longer operative duration, higher blood loss, and decreased mechanical strength of the plate. Additionally, the proximal locking screws in these plates may not achieve the “raft” effect to support the articular surface of the posterolateral tibial plateau fracture. Therefore, details of an innovative anatomic locking plate for the posterolateral tibial plateau (Fig. 1) and the clinical outcomes were reported to evaluate the safety and clinical efficacy of this innovative plate.

## Methods

### General data

This retrospective study was conducted after obtaining the approval of the institutional review board. Between March 2014 and January 2016, 12 patients with a posterolateral tibial plateau fracture (ages 18–45 years) underwent surgery with the anatomic locking plate for the posterolateral tibial plateau. Patients with osteofascial compartment syndrome, open fracture, multiple trauma, pathologic fractures, autoimmune diseases, blood disorder, and surgical contraindications were excluded. All patients received anteroposterior and lateral radiographs and three-dimensional computed tomography reconstruction images of the tibial plateau before surgery. Additionally, all patients were followed up either by clinic review or by telephone as required.

According to the CT reconstruction images, the main fragments were located in the posterior half of the lateral condyle and/or a fracture line impacted the posterior aspect of the lateral plateau. According to three-column classification [10], all cases were fractures of the posterolateral column. Preoperative management included distal bony traction and splint. After the soft tissue condition was stable, all the fractures were exposed and reduced via the posterolateral approach by the same surgeon (Yu).

### Plate details

**Fig. 2:**
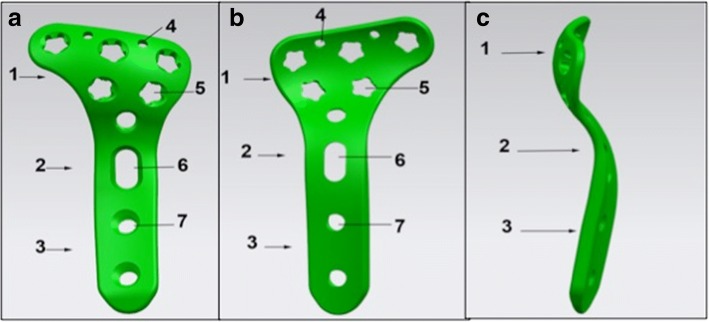
Schematic figureof The newly designed posterolateral anatomic locking plate. **a**, posterior view; **b**, anterior view; **c**, lateral view(1.head, 2.waist, 3.body, 4.K-wires hole, 5.locking screw hole, 6.sliding compression hole, 7.locking screw hole)

The anatomic locking plate for the posterolateral tibial plateau (Fig. 2) was designed according to the structure of the posterolateral tibial plateau in a Chinese population. It was oblique “T” shaped to fit the posterolateral structure of the tibial plateau with a low profile and beveled edges. The angle between the plate head and the waist was 136°. It had five 30° universal locking holes to ensure precise screw insertion in the head, which fixed the plate with the posterolateral side of the tibial plateau. Especially three proximal row screws paralleled with the plateau could buttress powerfully as a “raft effect.” Tiny holes in the top of the plate head were used in cases of a temporary fixation or comminuted fragment fixation by K-wire insertion and capsule sutures. When plate data was transefer into 3D computer-aided design (CAD) model, several patients’ knee CT data were also integrated with the CAD model, implant placement was simulated in silico to check for accuracy. When we obtained the plate patent, the plate was made by a qualified custom-made plate manufacture (WASTON Medical Appliance Co, Ltd., China). All structure was made of titanium. Finally, the plates obtained were completely consistent with our design in terms of their shape and distribution and the orientation of screws.

### Surgical technique and rehabilitation

All operations were performed using spinal anesthesia or general anesthesia with the patients in the prone position, and the lower limb tourniquet was inflated.

A posterolateral incision was placed over the biceps femoris muscle. Attention must be paid to identify and dissect the peroneal nerve posterior of the biceps femoris muscle. Then gently retracted laterally for the nerve protection during the operation. Medially retracted the lateral head of the gastrocnemius with the origin of the soleus elevated from the proximal section of tibia. The popliteus tendon could be retracted proximally to allow for fracture site visualization. The soleus was then detached from dorsal fibula with carefulness. Thereafter, the exposed capsule was incised to allow for the visualization of the entire lateral aspects of tibial plateau, including the posterolateral corner.

Temporary fix the fracture fragments with multiple K-wires. After elevation, the defect was filled with artificial bone graft. Then, screw formation of the posterolateral anatomic locking plate was attempted. Three “raft” proximal row locking screws were inserted parallel with the plateau joint surface. The reduction quality and location of internal fixation were further identified by fluoroscopic guidance. The physical examination of the knee stability was routinely conducted to exclude ligament injury.

Mobilization of the knee joint was encouraged immediately after surgery, and discharge was agreed when 90° was achieved, usually 5–10 days postoperatively. The knee motion range, as well as muscle strengthening was increased after surgical incision healing. Patients were initially allowed no weight bearing for 12 weeks to avoid early loss of reduction. Depending on radiographic signs of healing, partial weight bearing was advised and advanced to full weight bearing as soon as the patients can tolerate.

**Fig. 3:**
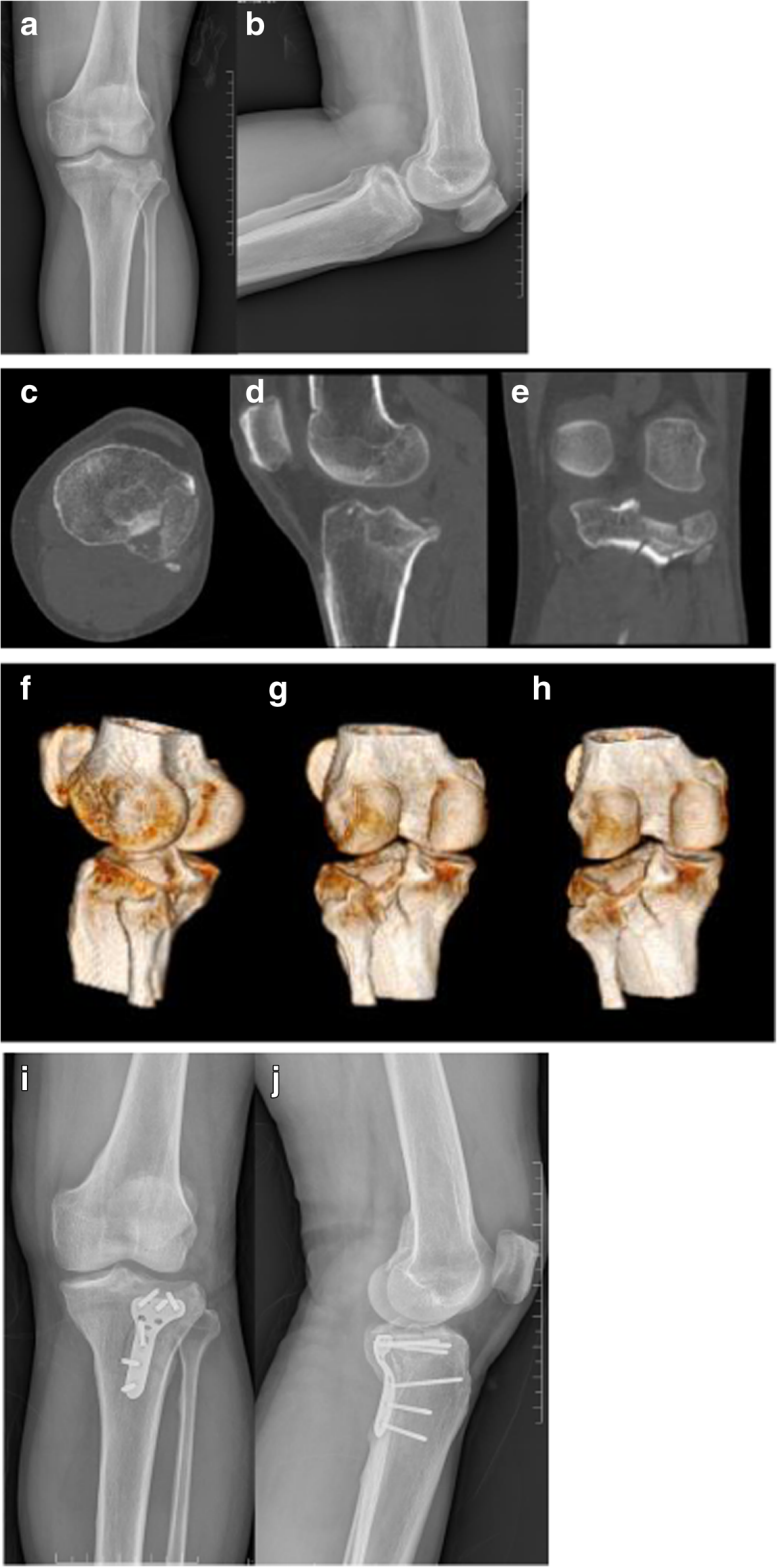
One male patient with posterolateral tibial plateau fracture, 43y, caused by car-accident. **a**, **b** were preoperational anteroposterior and lateral radiographs of one posteroleteral tibial plateau fracture. **c**-**h** showed more fracture details by 3-D reconstruction of CT scan. **i**, **j** were anteroposterior and lateral radiographs of the patient acquired after open reduction and internal fixation with the plate

Clinical evaluation was performed using the Tegner–Lysholm functional score, Rasmussen functional score [4], and Rasmussen anatomical score [4]. The Rasmussen functional score was used to evaluate knee function primarily with special attention paid to the single plane stability and rotation stability. The Rasmussen functional score is a specific outcome index for postoperative evaluation of tibial plateau fractures. It consists of five categories, including pain (6 points), walking capacity (6 points), extension (6 points), total range of motion (6 points), and stability (6 points). The total score is the sum of these five items, and the higher scores indicate the greater function. A score of 30 points is defined as normal, 27–30 as excellent, 20–26 as good, 10–19 as fair, and 6–10 as poor. The Rasmussen anatomical score consists of three categories, including depression (6 points), condylar widening (6 points), and angulation (6 points). A score of 18 points is defined as excellent, 12–17 as good, 6–11 as fair, and 0–5 as poor. Medical image data of one patient is shown in Fig. 3.

## Results

The average follow-up was 26 months (12–34 months). The average age of patients was 35 year-old (18–45 years). The general conditions of patients are listed in Table 1. The mean time from injury to surgery was 6.1 days (0–12 days). At 12 weeks, evidence of radiological fracture union was obtained in all patients (10–14 weeks).

**Table 1:** General conditions

Case Number	Age(y)	Sex	Cause	Follow-up(mo)
1	18	Male	Veh.Acc.	12
2	43	Male	Veh.Acc.	18
3	42	Female	Fall	32
4	38	Male	Fall	22
5	45	Male	Fall	20
6	41	Female	Veh.Acc	24
7	39	Male	Fall	34
8	35	Male	Fall	31
9	33	Male	Veh.Acc	28
10	36	Female	Fall	33
11	33	Male	Fall	27
12	31	Female	Struck	31

During surgery, time and blood loss were recorded. No case of ligament reparation was found in the study because all patients with knee instability were excluded. Nine patients obtained anatomic articular surface reduction as well as three obtained good reduction.

Tegner-Lysholm function score, Rasmussen functional score, and Rasmussen anatomic score of final follow-up were recorded (Table 2). Radiograph showed intra-articular reduction loss of 2 mm step-off in one case, which might be caused by weight-bearing too early. No knee instability or pain was found in all cases. The average knee motion arc was 137° (122°–153°).

**Table 2:** Clinical findings

Case Number	Blood loss (ml)	Operation duration (min)	Tegner–Lysholmscore	Rasmussen functional score	Rasmussen anatomical score
1	250	70	97	29	18
2	100	55	93	28	16
3	160	55	87	27	14
4	200	60	89	28	15
5	230	70	85	25	13
6	200	65	95	26	16
7	210	80	90	26	15
8	240	80	91	28	16
9	150	75	93	27	16
10	300	90	88	28	15
11	180	75	88	27	16
12	220	70	89	26	15

Incidence of CPN (Common Peroneal Nerve) injury was noted in none case. Lateral knee instability was seen in none case. No flexion contractures or extensor lag was seen. No deep vein thrombosis or graft site morbidity was seen at the follow-up. In addition, no case of incurred nonunion or internal fixation failure was reported. However, one patient had superficial wound infection that recovered after treatment with antibiotics and local dressing changes.

## Discussion

Patients with untreated posterolateral tibial plateau fracture may suffer from chronic pain, knee instability, and loss of function, especially when climbing the stairs, which even leads to malunion that needs surgery or re-surgery. Thus, many surgeons have agreed that open reduction and internal fixation should be preferred over non-operative treatment [1, 2, 4, 5]. Multiple approaches have been described given the complexity of the posterolateral tibial plateau anatomy [2].

Lobenhoffer et al. [9] introduced a posterolateral approach with fibular osteotomy with better visualization. An anterolateral approach with partial or full fibula head removed was developed by Yu [4], which do great benefit for reduction and fixation in the treatment of posterolateral tibial plateau fractures. However, fibular osteotomies may injury the common peroneal nerve and decrease the stability of the lateral knee joint [11]. Frosch et al. [8] described a posterolateral approach with a lateral arthrotomy for visualizing the joint surface. Posterolateral approach was modified by Tao [7] with an L-shaped incision. Fibular osteotomy was not required in this approach and it protected the soft tissue well, showing encouraging clinical outcomes. The posterolateral approach without fibular osteotomy was chosen in the present study.

Although surgical approaches have been well discussed in previous studies [4, 7–9], operative fixation in treatment of posterolateral tibial plateau fractures has rarely been reported. No specialized plate is used for the treatment due to the irregular shape of the posterolateral tibial plateau. The most widely used system is the distal radius locking plate [1]. The plate usually has to be contoured during surgery to achieve satisfying fitness, which may damage the locking hole and affect the angle stability. Moreover, the plate cannot achieve the “raft” effect due the fact that the proximal locking screws are parallel to the joint surface. This may lead to a higher risk of reduction failure, further comprising outcomes.

Cho et al. [3] reported a novel technique of 2.7-mm rim plating in tibial plateau fractures to address these problems. Rim plates function as positional plates (named “Hugging plates”) rather than buttress plates. The rim plate maintains the posterolateral tibial fracture reduction with the collinear clamp used for a rigid maneuver reduction throughout the fixation procedure. This rim plating technique can be easily applied to isolated posterolateral fracture fragments. However, it is difficult to use in patients with posterolateral tibial comminution and posterolateral corner depression. Moreover,both anterior and posterior stripping to obtain an adequate operative field, together with less antiskid ability, limits its use.

The newly designed anatomic locking plate for the posterolateral tibial plateau, which is oblique “T” shaped, fits the posterolateral structure of the tibial plateau well enough that it reduces plate contouring during surgery, shortens operative duration, decreases blood loss, and potentially decreases plate failure. In our study, our new anatomic locking plate fit anatomically, requiring no additional manipulation. The beveled edges and low profile of the plate can reduce the risk of prominence, which always leads to secondary implant remove surgery. Further, the proximal row locking screws can achieve the “raft” effect, which provides firm strength holding the articular surface. The main limitation distally is the posterior tibial neurovascular bundle. It is difficult to safely achieve exposure beyond approximately 8–10 cm distal to the lateral joint line owing to vessel trifurcation that transverse the interosseous membrane and muscle mass medial to the fibula. However, the length of plate is appropriate to reach the distal border, …without damaging the neuromuscular bundle. Thus, distal tibial exposure is not necessarily required. According to the Rasmussen functional/anatomical score, all patients achieved “good” results. The plate was proven to be safe and effective in a small-sample-size population (12 patients) during a 12- to 34-month follow-up.

The limitations of the study were the relatively small sample size; hence, large-sample studies need to be conducted to prove the efficacy of the technique. In addition, further biomechanical studies are required to evaluate buttress performance, compression forces, and comparative features against traditional buttress line plates via the posterolateral approach.

## Conclusions

In conclusion, the newly designed anatomic locking plate for the posterolateral tibial plateau was proven to be safe and effective in a small-sample-size population (12 patients) during a 12- to 34-month follow-up. The plate fit the irregular posterolateral tibial plateau well, without requiring additional contouring. Therefore, this new plate may be used as a favorable internal fixation choice for treatment of posterolateral plateau fractures.

## Abbreviations

CPN: Common Peroneal Nerve
CT: Computed tomography
